# The Gradual Transformation of the Polish Public Science System

**DOI:** 10.1371/journal.pone.0153260

**Published:** 2016-04-14

**Authors:** Steffi Heinecke

**Affiliations:** Institute of Sociology, School of Human and Social Sciences, University of Wuppertal, Wuppertal, Germany; Utrecht University, NETHERLANDS

## Abstract

This paper investigates institutional change in the Polish public science system (PPSS) in the past twenty years. Employing macro-statistical data, the paper argues that this change process has unfolded stepwise and relatively late despite major political and economic transformations in post-socialist Poland. Using a historical-institutionalist perspective, the paper focuses on processes of institutional change, including layering, displacement, and dismantling. One major finding is that the speed and depth of the gradual transformation differs considerably between the three research performing sectors of the Polish public science system. As the Polish Academy of Sciences was reproduced institutionally, the former governmental units for applied R&D were partly dismantled and displaced by private sector R&D units. In contrast, the Higher Education sector underwent a strong expansion and, thus, layering of new research activities and fields. Since policy shifts within the PPSS occurred relatively late, the more than two decades following the collapse of communism are of special interest to scholars of incremental, yet cumulative, institutional change.

## Introduction

Studies focusing on transformations in post-socialist countries have usually dealt with major economic and political shifts, particularly regarding democratization, civil participation, and the transition from formerly planned economies into market economies [1], [2], [3], [4]. Though these studies offer explanations for the political and economic transformation of Poland, the literature has been relatively silent regarding other societal sectors, such as science and innovation. Therefore, this paper adds new insights about institutional change in the Polish public science system (PPSS). Following studies on the institutional character of science, a public science system is understood as “the set of organizations whose employees undertake research primarily for publication together with the institutional arrangements governing their operation, including their funding, establishment of priorities, evaluation of performance and allocation of rewards.” [5] Like many other post-socialist public science systems, the PPSS has not attracted due attention in the transformation literature, mostly because it did not undergo the same discontinuous transformations that the political and economic sectors witnessed immediately following the peaceful revolution in 1989.

In contrast to the democratization and market liberalization of post-socialist societies, the PPSS appears to be rather stable, despite the political and economic turmoil of the late 1980s and early 1990s; institutional change has unfolded stepwise and relatively late.

One striking feature of the PPSS is the threefold structure of its performer level, which was inherited from state socialism: the *Higher Education sector* (HE), formerly dedicated solely to teaching; the *Polish Academy of Science* (PAS), responsible for basic research; and the *Governmental Research and Development Institutes* (GRDIs), which are in charge of applied research.

In the forty-five years of communist rule, Polish science took place within these three sectors. After 1989, business enterprises and private non-profit actors set up research and development units and they contribute to Polish science today. In 2010 however, the three above mentioned sectors accounted for seventy-seven per cent of all scientific personnel (nineteen per cent were employed in economic entities) and seventy-three per cent of all expenditures on R&D were spent in the institutes of HE, GRDIs and PAS. When considering the sources of funding, we find that in 2010, sixty-one per cent of all R&D expenditures stemmed from the Polish government and only twenty-eight per cent from the private sector. In many Western countries, this ratio is the opposite: one-third of the national R&D budget comes from the national government and two-thirds from the private sector [6], [7]. The analysis presented in this paper therefore focuses on the three mostly publicly funded sectors which were created in the beginning of the Polish People’s Republic (PRL) and still constitute the central part of science in Poland.

The legal acts passed in the immediate aftermath of the collapse of the PRL, particularly the *Act on Higher Education and Scientific Titles and Degrees* (September 1990) and the *Act to Establish the State Committee for Scientific Research* (January 1991), and the subsequent reforms and amendments did not have direct and major effects on this sectoral structure. Rather, the early legislation established the principles of freedom and democracy at universities, allowed the foundation of private schools, and granted administrative and organizational autonomy to institutes of higher education. Additionally, the legislation established a new actor, the *State Committee for Scientific Research* (KBN), which was partly elected by the Polish scientific community and partly appointed by the government. Responsible for budget proposals, the allocation of funds, and the development of evaluation criteria, the KBN embodied the newly established autonomy of the scientific community. At the same time, the KBN resembled the centralized structure of the Socialist system by concentrating the role of the policy maker and the funding agency in its dual function as a research council and a ministry-like body. Like some of the other structural features of the PPSS, the centralized funding mode was not changed by the new laws. Although the former legal structures were mostly abolished in 1990, some key structural features as well as the organizational structure of the PPSS were left untouched.

Sudden liquidation of research institutes or vast reduction of staff did not occur in Poland as it had in other Central Eastern European Countries (CEECs). The persistence of institutional and organizational structures despite shifts in public policy has been observed in other sectors of post-socialist societies as well [8] [9]. It seems that the transformation processes oftentimes take place within the existing institutions inherited from socialism. Therefore, it is necessary to shift the attention from the policy changes towards these structures and the ongoing processes of change within them.

The existing literature, however, has mostly focussed on a) the policy level including legal reforms, policy shifts and important actors and b) the early stages of PPSS’s transformation [10] [11] [12]. Consequently, these studies put a focus on processes of radical change and assumed the *breakdown and replacement* of the socialist science system [10] [13]. By considering a) the changes in organizational structures and resources available to research performers and b) tracing these over a longer period, this paper offers a new perspective.

It applies a historical-institutional perspective to capture the rather slow-moving processes that occurred over the first twenty years of post-socialism. This period is marked at its beginning by the collapse of the PRL in 1989/90, and ends with the major reforms in the science system in 2010 and 2011. Previous studies have already analysed policy changes and the actors involved [14], [10] [11], [15], the effects of external forces like Europeanization [16], [17] and the specifics of the Higher Education sector [12], [16], [17]. While these accounts are extremely enlightening, this paper strives to add another perspective to the study on post-socialist public science systems. Using macro-statistical data, it maps out the institutional setting, in which scientific research has taken place in the past twenty years. The dynamics within the organizational structure of the research performing sector are first presented in quantitative terms (Results) and later put into context (CDiscussion).

Beforehand, the theoretical approach of historical institutionalism is introduced and how this approach fits the research question of this article explained. Altogether, the paper attempts to capture the processes unfolding in the PPSS over the last twenty years by focusing on how gradual, yet cumulative, changes have transformed the post-socialist public science system in Poland.

## Theoretical Approach

Within the studies of institutional change, different answers have been provided to the question of how and why institutions change. This section discusses two prominent concepts in light of post-socialist transformation: the concept of *path dependency* and the concept of *gradual transformation*. The former conceives of change as the abrupt departure from a historical path after an exogenous shock that ultimately leads to a new institutional equilibrium, whereas the latter understands change as a process of incremental steps that gradually transform an existing institutional arrangement.

Path dependency was developed in studies of technological change that explained why ineffective technologies prevail over more effective technologies [18], [19], thereby challenging neo-classical assumptions. After being adopted for the field of comparative politics [20], [21], the concept’s prominence in social sciences was elevated in the early 2000s with publications by Pierson and Mahoney [22], [23]. Focusing on the reproduction of institutions and self-reinforcing processes leading to inertia, studies of path dependencies cover institutional change mostly at the beginning of a path, and sometimes at the end of a path. Change seems rather unlikely once a certain path is “locked-in”.

The lock-in argument was picked up by Kathleen Thelen, who argued that a binary distinction between institutional change and institutional stability masks the fact that mechanisms of reproduction and mechanisms of change are often at work concurrently [24], [25]. Her main critique is that the concept ‘is premised on a punctuated equilibrium model that emphasizes moment of openness and rapid innovation followed by long periods of institutional stasis or “lock-in”‘ [25].

One prime empirical example of such a moment of openness and the possibility for rapid change is the collapse of state socialism at the end of the 1980s. Early comparative studies in the post-socialist transformation rested on the assumption that the demise of state socialism left an institutional vacuum in the CEECs, which opened a window of opportunity for domestic and foreign actors to install Western institutions of the free market and a democratic political system. As ‘normal constraints of social structures and political institutions seem temporarily suspended’ [1], several studies ignored the embeddedness of institutions in a broader social context [26]. However, the crisis of both the political and the economic system at the end of the 1980s did not lead to a complete dismantling of socialist institutions.

In light of that, a second approach was developed that stressed the institutional, structural, and cognitive resilience of socialist legacies over the course of the transformation [27], [3], [4]. Contradicting the neo-classical perspective, this approach assumed that the ‘transformation will resemble innovative adaptations that combine seemingly discrepant elements−*bricolage*−more than architectural design’ [28]. Most authors working with the concept of path-dependency in the post-socialist context viewed the impact of socialist legacies as the confinement of strategic choices made in the early stages of the transition. The existing institutions affect the choice of a certain path, and its further development is shaped by increasing returns, adaptive learning, and the avoidance of transaction costs for institutional change leading to a departure from this path. Thus, most studies focus on the immediate transition rather than the subsequent developments in post-socialist societies. By limiting the concept of institutional bricolage to the constriction of choices made in the early stages, the various ways in which institutions inherited from state socialism affect the transformation are not accounted for. How some of these institutional elements are reproduced, reused for different purposes, complemented by new elements, or simply vanish cannot be understood properly when following the path-dependency approach.

Following this line of argument, Thelen challenged the notions of both institutional stability and exogenously generated institutional change that constitute the main assumptions of the path-dependency literature. Since institutions are understood as “formalized rules that may be enforced by calling upon a third party” [29], only changes to these formalized rules–like new policies or legislation–are commonly considered as institutional change. Thelen and Streeck have broadened our understanding of institutional change by investigating different types of change. These are displayed in the following typology and do include more than just changes in the formal rules (see Table 1).

**Table 1:** Types of institutional change [29].

		Result of change	
Process of change	Incremental	Continuity	Discontinuity
		*Reproduction by adaptation*	*Gradual Transformation*
	Abrupt	*Survival and return*	*Breakdown and Replacement*


Institutions are viewed as constantly changing because the formal rules they embody need to be a) enacted and interpreted constantly and b) realigned with changing social, political, and economic conditions. Within the processes of a) enactment through interpretation of a formal rules by actors and b) adaptation to changing environments, informal practices develop that may differ from the formal rules. While these informal practices ensure the functioning of the institution by aligning it with its environment, they lead to gaps between them and the formal rules. Over time, these gaps become so big that the formal rules need to be adjusted in order to match the informal practices–this is what the authors call gradual, endogenous change.

Thelen and Streeck have shown that most institutions in Western welfare states are able to survive over a long period because they change over time, not abruptly after a shock in their environment, but incrementally [29]. Five modes of gradual transformation have been specified by the authors in order to understand the processes of endogenous change [29]: (1) *displacement*−existing rules and/or structures are removed and replaced by new ones; (2) *layering*−existing rules and/or structures are kept in place and new ones are added on top; (3) *conversion*−existing rules and/or structures are redeployed for new purposes through changed interpretation or enactment; (4) *drift*−existing rules and/or structures have a different outcome/impact due to changes in the environment; (5) *exhaustion*−existing rules and/or structures dwindle away as they are misused or overextended. A sixth mode was added in studies focusing on the renewal of research organizations: *dismantling*−existing rules and/or structures are dismantled and no new ones installed [30], [31]. These processes take place beneath the policy level. They are not the product of intended laws or policies, they occur within the institutional and organizational structures while the formal rules are a) enacted and b) realigned with changing environmental conditions. That is why it is necessary to investigate not only the policy level, where formal rules are conceived and adopted; but also the institutional and organizational structures, where these formal rules are enacted and realigned.

Most previous studies on transformations of science and innovation systems in CEECs have typically focused on the policy level only. They assumed *breakdown and replacement* for the public science systems and have paid only little attention to the institutional and organizational legacies of socialism [10], [13]. However, a few studies have already adopted a historical-institutional perspective while tracing the development of public research funding systems in CEECs [11], [12]. They have shown that, regardless of the dramatic changes in Poland after 1989, change within the PPSS has occurred rather evolutionary.

Still missing from the literature on the post-socialist PPSS is the performer-level. There are no instructive studies on the research performing sectors and their de facto development after 1989. Since the concept of institutional change includes formal rules (laws, regulations, policies) as well as organizational structures (organizational units, staff positions, monetary resources), it is important to complement the study of research policies by a study of organizational structures on the performer level. In order to do so, this paper presents longitudinal data on the organizational structure of the research performing sectors of the PPSS.

As the data presented below shows, no radical reduction in resources occurred after new laws were introduced in 1990 and 1991. In contrast, modest cutbacks in staff, operations, and investments were matched by the preservation of most research units in the early stages of transition. Changes in its formal rules did not immediately affect the organizational structures of the PPSS. In post-socialist Poland, these structures include three sectors of research performers that differ with regard to their function during socialism. The reforms introduced in 1990/91 did not touch the organizational structure of the three sectors or decrease the organizational segmentation. Neither did the reforms tackle other structural features of the PPSS, which were inherited from Socialism: like collegial governance modes and vested interests of the scientific community, complicated multi-level system of academic career, a lack of transparency and mobility in the labour market for scientists, or relatively non-competitive and centralized funding modes [11], [12]. How these various features have interacted with the reforms of 1990/91 and shaped the development of the PPSS has been explored elsewhere [11], [12].

This study focusses on the organizational structure of the PPSS and the dynamics that unfolded over twenty years within the three research performing sectors. The longitudinal statistical data presented ihere shows that the reforms in 1990/91 did not lead to an immediate restructuring. Instead, the processes of change unfolded gradually in the face of sector-specific environmental changes. Therefore, it seems fruitful to apply Thelen and Streeck’s conceptual scheme that puts cumulative change in the organizational and institutional structures over an extended period of time at the centre of the analysis, rather than focusing on radical shifts on the policy level.

## Methods and Materials

The aim of the paper is to map the PPSS with special regard to the three sectors over the course of the past twenty years. As of today, there is no comprehensive longitudinal statistical analysis of the Polish research system, thus the effects and causes of policy changes have not been traceable on that level. This paper offers indicators for all three sectors which are suitable for a valid comparison. To this end, the data was transformed into data sets which provide comparable categories, inflation adjustment and recognition of broader trends by using three-year moving averages. The first data set consists of the number of entities conducting research and development (R&D units) in socialist and post-socialist times, covering almost forty-five years. The second relevant data set consists of the staff numbers for the R&D sector in Poland, expressed in full-time equivalents (FTE), covering the past twenty years. The third data set relates to expenditures on research and development. Converting expenditures in the 1980s and early 1990s is practically impossible due to currency instability [32]. Therefore, inflation-adjusted absolute numbers are presented for R&D expenditures since 1994 relying on the gross expenditure on R&D (GERD) and the gross domestic product (GDP) as references for a comparison of the socialist and post-socialist science system. With regard to institutional continuity, the sources of funds for R&D and their development over time are of interest, including the state budget, business sector, R&D performing institutes, and international organizations.

The statistical data were retrieved from various statistical reports provided by the Central Statistics Office of Poland (Główny Urząd Statystyczny, GUS), which adapted the criteria codified in the OECD methodological handbook, known as the Frascati Manual, in 1994. Most of the data was taken from the statistical yearbook (Rocznik Statystyczny) and the yearly reports on science and technology (Nauka i technika, continuously available since 1992). As OECD criteria were only introduced in 1994, data from earlier years are to be handled with caution–the data from different reports during socialist times vary significantly.

Indicators of scientific output are not included in this analysis. Due to the complex nature of the relation between research input (funding, staff, organizational entities) and scientific output [33], this aspect is left for future analysis [34].

Additional background data were taken from various legal acts and KBN documents, including official journals and individually passed regulations. Regulations and strategy papers published by the Ministry of Science and Higher Education were taken into consideration, as well as discussion and position papers published in monthly publications and various publications of the PAS.

## Results: Macro-statistical Comparison of the Three PPSS Sectors

The units represented in the data below include the scientific institutes of PAS, GRDIs, and HE. In this section, macro-statistical data are used to outline the developments in the three sectors with regard to the number of units performing R&D, scientific staff, and funding.

**Fig 1:**
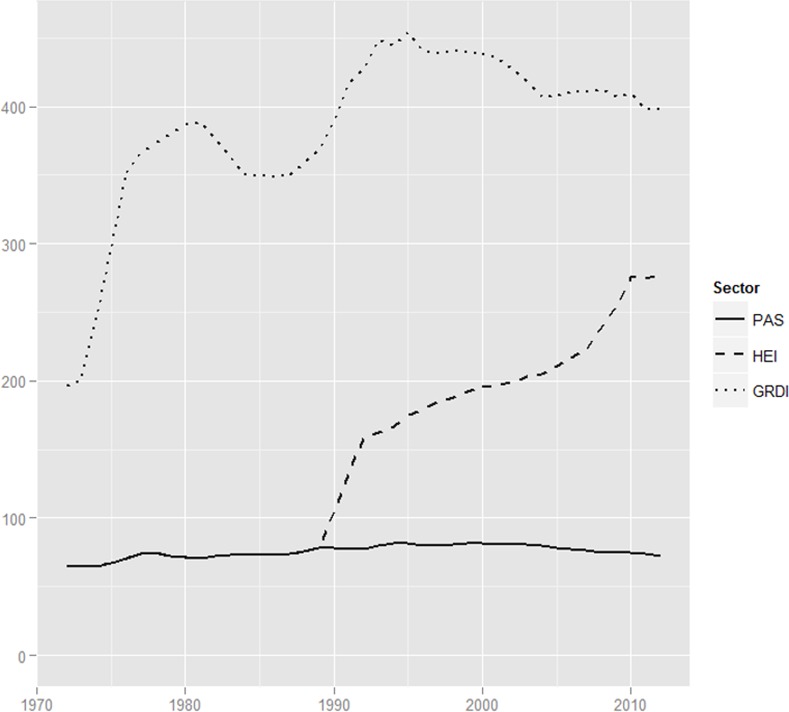
Number of R&D units according to PPSS sector; data displays cumulative, three-year moving averages of the number of R&D units.

Fig 1 shows the number of units performing R&D over the past forty-five years. The number of GRDIs has declined substantially, with certain peaks in the early 1990s. This decline started in the 1980s due to the major economic crisis in Poland and continued after the collapse of state socialism in 1989. From 322 institutes in 1980, to 260 in 1990, the number fell to 211 in 2002 and eventually to 119 in 2012. Therefore, more than sixty per cent of all GRDIs were closed during this period, thereby reducing a substantial part of the R&D capacity of GRDIs. With existing units being shut down and no new units performing equivalent tasks being built up elsewhere, the GRDI sector has witnessed a *dismantling* over the past twenty years.

In sharp contrast, the number of HE institutes has grown considerably. Although, before 1989, no separately accounted research institutes were present at universities; the HE institutes in Poland conducted research [16]. Therefore, the number of new research institutes in 1990 was already eighty and grew to over 200 in 2012, which is equivalent to a growth rate of 2.5. A growth rate like this is typical for a process of layering, which is propelled by differential growth. New structures or practices are introduced that “operate on a different logic and grow more quickly than the traditional system (so that) over time they may fundamentally alter the overall trajectory of development as the old institutions stagnate or lose their grip and the new ones assume an ever more prominent role in governing individual behaviour.” [29] As the number of HE institutes increases and more resources are allocated towards them, the other two sectors receive less resources and undergo decline. Thereon, the HE institutes are able to attract more funding and staff (see below)–thereby gradually transforming into *the* research performing sector of the post-socialist PPSS.

During Socialism, the majority of basic research was performed by the PAS institutes. Their number has been relatively stable over the whole period. As most PAS institutes were founded in the 1950s and 1960s and have survived until today, only slight deviations have occurred over the decades due to mergers or occasional closures of institutes. From seventy-seven research institutes in 1990 to eighty-one in 2000 and seventy-six in 2010, the decline is mostly due to restructuring and variations in counting.

**Fig 2:**
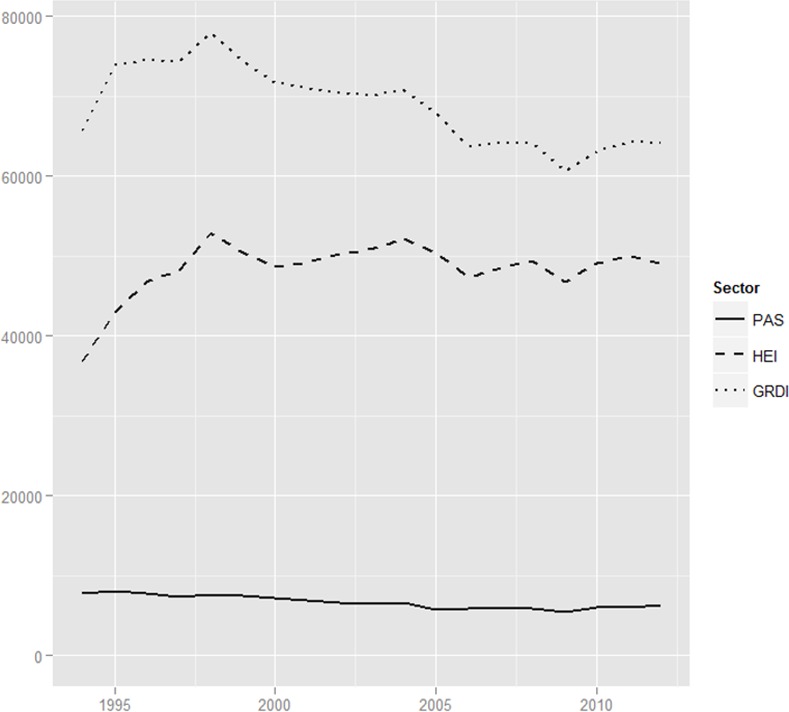
R&D staff, according to PPSS sector, shown in Full Time Equivalents (FTE); data displays cumulative, three-year moving averages.

In the second data set, the R&D staff numbers include researchers, technicians, and equivalent staff, as well as other supporting staff, all of which are ‘employed directly on this activity (or providing direct services for R&D) and spending at least ten per cent of their nominal working time on R&D’ [35]. Beginning again with the GRDIs, Fig 2 shows that they started with approximately 29,000 R&D staff in 1994. This number was cut in half by 2012. This reduction is clearly connected to the shut-down of institutes and partly due to early retirements and special bonus programs that encouraged people to leave. The qualification structure is also important, as the reduction was not made so much within the scientific staff, but mainly targeted supporting staff, including skilled and unskilled craftsmen, as well as secretarial and clerical staff. In summary, the reduction of R&D staff at GRDIs was strongly connected to the dismantling of GRDI units. Concurrently, the rapid growth of research institutes in HE since the mid-2000s is reflected in an increase in R&D staff. The number in R&D staff at HE institutes increased from approximately 29,000 in 1994 to 43,000 in 2012 (see Discussion), which is equivalent to a 65 per cent increase in R&D staff, which is less than the strong increase in HE units but nevertheless reflects the strong *layering* process that occurred in the HE sector over the past twenty years.

The R&D staff at PAS institutes decreased from approximately 7,800 in 1994 to 5,450 in 2009, which is equivalent to a staff reduction of roughly thirty per cent over fifteen years. Notably, this reduction, which affected mostly the non-scientific staff, stopped in 2009; since 2010 the staff numbers have increased to 6,300. During the whole period, the reduction in staff suggests a dismantling pattern at PAS institutes.

**Fig 3:**
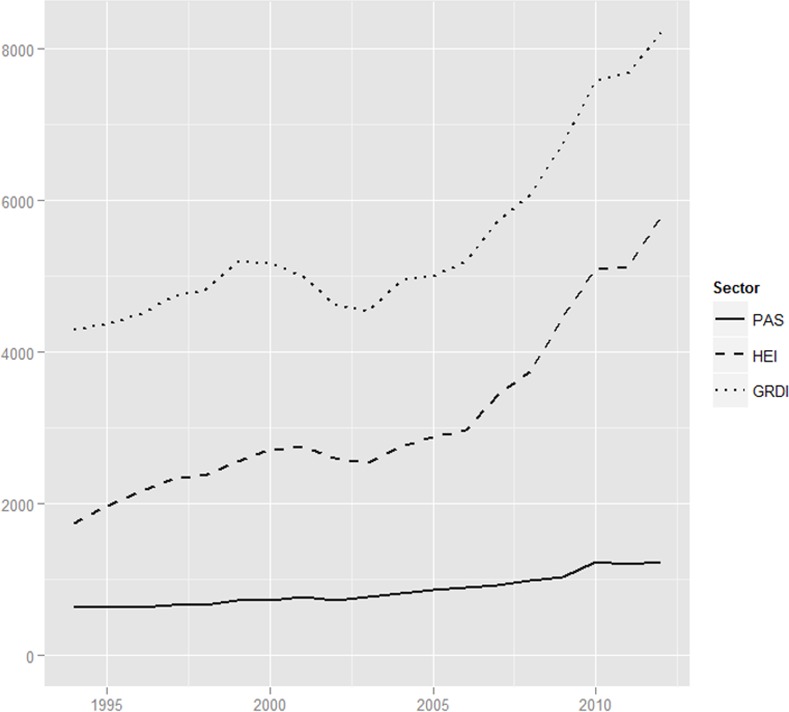
Gross domestic expenditures on R&D in million PLN according to PPSS sector, inflation adjusted data, base year 2010 (CPI taken from OECD statistics); data displays cumulative three-year moving averages.

In the third relevant data set, the slight decrease in the GRDI R&D budget suggests that, given the substantial reduction of both institutes and R&D staff, the remaining GRDIs had relatively more resources at their disposal and were better equipped than prior to 1990 (see Fig 3). A shrinking (inflation-adjusted) budget was observed in the first ten years (from 2,560 million PLN in 1994 to 2,000 million PLN in 2003), but the past ten years have seen a slight increase (to 2,400 million PLN in 2012). In summary, the dismantling process, which could be observed at the level of GRDI units and GRDI staff, was less pronounced in the budget dimension.

The foundation of new HE institutes is clearly reflected in the increased R&D budget (4.5 times), with an enormous increase since 2008: 1,100 million PLN in 1994, 2.5 bn PLN in 2007, and 4.6 bn PLN in 2012 (see S3 Table for complete data). Most importantly, however, compared to PAS and GRDI, HEs share in the total national R&D expenditure grew from twenty-five per cent in 1994 to fifty-one per cent in 2011. Therefore, the R&D budget data strongly supports the overall conclusion that HE institutes have undergone a *layering* process in the past twenty years.

The PAS has seen a steady growth of R&D expenditures, from 646 million PLN in 1994 to 1.2 bn PLN in 2012 (see Fig 3), with a relatively stable number of institutes, suggesting that these institutes are now better equipped. The doubled R&D budget of the PAS may have led to the layering of new research fields in the research institutes over time and requires more in-depth study but is beyond the scope of this article.

In summary, with regard to institutional change processes, the three data sources are largely consistent. The dismantling of GRDIs seems to be profound in regards to the shut-down of institutes, the reduction of R&D staff, and the decrease in funding. Interestingly, the R&D budgets of the PAS and GRDIs appear to be somewhat decoupled from the closure of institutes and the reduction in R&D staff. This may indicate processes of layering in other fields in the remaining PAS and GRD institutes, but it may also mirror the fact that R&D has become much more expensive over the past twenty years. Meanwhile, the broad pattern of expansion and layering observed for HE institutes for all three indicators stands in stark contrast to the overall pattern of decline for the GRDIs and apparent stability of the PAS. The considerable reduction in R&D staff until 2009 at PAS institutes provides evidence that a *dismantling* process has occurred over the past twenty years. However, the doubled R&D budget suggests the possibility of incorporating new research fields into the remaining PAS institutes. As HE is the only sector in which substantial growth can be observed, the contrast between *dismantling* (GRDI), *relative stability* despite R&D staff reduction (PAS), and overall *layering* (HE) calls for an explanation, which is provided in the next two sections.

## Discussion

After analyzing the processes of change in the organizational structure of the research performing sectors, it is clear that these differ from the changes on the policy level in speed and depth. Jablecka has identified the following patterns of change on the policy level of the PPSS: “a phase of radical change in short time (1989–1991), a phase of substantial stability (1991), and finally a sequence of more gradual changes leading to further restructuring of research policy and funding (200–2007).” [11] The empirical analysis shows that changes on the organizational level were not as abrupt in the beginning; instead they unfolded over the decade of apparent “stability” and fueled the gradual changes since the mid-2000s. An explanation for this discrepancy is offered by the concept of gradual transformation. It stresses that a change in the formal rules (radical reforms 1989–1991) need to be a) enacted and b) realigned with changing conditions in the social, political and economic environment. The following section traces the processes of gradual transformation as a) the enactment of formal rules and b) the adaption to changing environment, which occurred in the form of dismantling, layering and reproduction by adaptation, for each of the three sectors.

### Economic Decline and Privatization: Dismantling GRDIs

Two explanations address why so many GRDIs were dismantled. First, Poland experienced a deep economic crisis after 1989. Although the decrease in R&D staff in GRDIs had already started in the early 1980s, it continued more rapidly after the 1989 crisis. For the first time GRDIs had competitors from the West, which could provide better and more cost-effective technological solutions and had higher qualified R&D staff. Therefore, GRDIs witnessed a significant decline in industrial demand for their R&D services. Before 1989, roughly thirty per cent of the R&D budget of GRDIs was external contract research, but these contracts were mostly discontinued in the early phase of Poland’s economic transition [36], [37].

Notably, during the first ten years after 1989, relatively few GRDIs were dismantled because most GRDIs received low but sufficient institutional funding from the Polish Government [15]. In 1990, almost seventy per cent of all GRDI funding was public statutory funding. However, within a few years, the level of statutory funding plummeted, falling to merely thirty-five per cent by 1995, whereas external R&D contracts had risen to sixty per cent [38]. Within five years, almost all GRDIs considerably changed the characteristics of the work they performed. R&D activities accounted for seventy per cent of the studied institutes’ activities in 1989 but decreased to approximately forty per cent by the end of the 1990s. GRDIs did less and less research, and more and more consulting, standardization work, and quality control.

The second explanation has to do with two amendments of the Act of 25 July 1985 on the Research and Development Units that became effective in 2000 and 2007. These amendments created new legal bases for the reorganization of ownership and organizational structure in order to enable GRDIs to adapt to the transition from a planned to free market economy. Many service-providing GRDIs were privatized, whereas the more research-oriented units were maintained as governmental entities. Over time, the function of the GRDIs in the economic system was increasingly taken over by the emerging private R&D sector. In 2005, there were 603 private R&D entities, in 2010 there were 977, and by 2012 their number had risen to 1,785 and employed ~23,000 staff, of which ~13,000 were researchers [36]. Therefore, many former state-owned GRDIs were replaced by new private R&D units.

In summary, the entire GRDI sector underwent institutional change via *dismantling;* in 1990 there were ~300 institutes, and this number shrank to 200 in 2003 and 119 in 2012 (Fig 1). Compared to other post-socialist countries, this dismantling did not occur radically and abruptly, but it took more than twenty years to shut down two-thirds of the GRDI units. As the number of R&D staff shows (Fig 2), the GRDIs were resilient against the enormous shifts in the sectors’ environment, at least in the beginning. Eventually, however, decreasing demand for R&D from state-owned research entities and regulations that helped a new private R&D sector to emerge were the main institutional factors that led to the gradual, yet cumulative, decline of this relict of state socialism in Poland, both in terms of research units and in terms of R&D staff.

### Demand for Tertiary Education: Layering New HE Institutes

As in the case of GRDIs, the transformative change in the HE sector has to do with changing conditions in its environment, including heightened demand for education, and new legal regulations. The end of state-socialism, not just in Poland, but also in other post-socialist countries, spurred new demands for *higher education*. Free access to tertiary education was an important component in the democratic transition of Poland. The *Act on Higher Education* was passed in September 1990 and organized HE on the principles of freedom and democracy, assigning various tasks to them, such as the education of students in the spirit of human rights and democracy and furthering scientific progress for the national well-being. The main focus of the 1990 Higher Education Act was to grant the organizations in the HE sector administrative and scientific autonomy.

Most importantly, the 1990 Higher Education Act made possible the establishment of private schools. Due to the rapid growth in the demand for higher education, these private HE providers were much needed and financially supported by the government. The number of students increased from 370,000 in 1989 to a peak of two million in 2005. Almost a third of all students were enrolled in private colleges and universities. The enormous demand by students led to a founding wave of new higher education entities. This created a situation in which it was possible for university researchers to accept multiple teaching positions, considerably reducing the amount of research conducted in HE [39]. Kwiek argues that during the 1990s and early 2000s, ‘teaching-related sets of organizational practice replaced previously well-established research-related sets of organizational practices’ [16] during these years.

The strong initial focus on teaching in the 1990s and early 2000s led to subsequent growth of research institutes at HE in the mid-2000s (Fig 1) and, accordingly, an increase in R&D staff (Fig 2). As the 2005 Law on Higher Education required all research-and-teaching staff and research staff to conduct research, the boom of HE units occurred when the expansion of teaching capacity in the HE sector reached its peak in the mid-2000s. Since the late 2000s there have been tendencies for the largely increased HE sector to enter a phase of consolidation and stratification [12].

**Fig 4:**
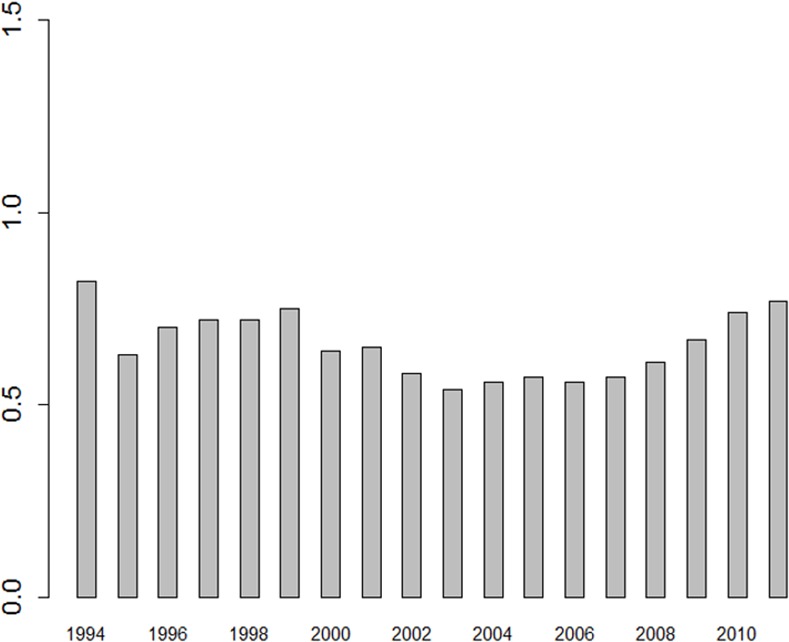
Ratio of R&D expenditures in percent of GDP.

Looking at the whole HE sector, the process of institutional change was clearly *layering*. The number of scientists employed in R&D units located in the HE sector increased from 29,000 in 1994 to roughly 43,000 in 2011. How many of these researchers were directly involved in research and to what extent cannot be quantified with the available statistical data. However, as the number of research institutes at HE increased from eighty in 1990 to 210 in 2012, a process of *layering* was definitely observed for research. Although layering appears to be difficult in the post-socialist environment due to low investments in R&D (Fig 4), the HE sector is an exception. The HE sector expanded considerably between 2006 and 2012, from 1 billion PLN to 4.6 billion PLN (inflation adjusted data, base year 2010); in the time same period sixty new HE institutes were founded. Therefore, most of the growth in the GDP ratio for R&D between 2006 and 2011 was channelled to the HE (Fig 4). As Kwiek pointed out, these investments were not evenly distributed over the HE sector, but there are tendencies of concentration and stratifications.

### Shielding Environmental Pressures from PAS via the KBN Funding Mechanism

Though the development of the GRDIs and HE was strongly affected by changes in the societal environment, the PAS was apparently unaffected by environmental factors, such as economic crisis (such as in the case of GRDIs) or strong demands for tertiary education (such as in the case of HE). The most obvious reason is PAS’ sole involvement in basic research, but two additional explanations are offered in this paper. The first explanation can be found in the continuity of legal regulations that grant the privilege of self-governance to PAS, and the second explanation has to do with the allocation mechanism by which PAS institutes were funded.

In its capacity as an umbrella organization of basic research institutes, the PAS is a truly socialist construction; founded in 1952, it is frequently referred to as the most socialist part of the PPSS [40]. As research programs were incorporated into the centrally-directed five-year plans set by the Communist party–with the basic research program set by the Scientific Secretary of the PAS–the PAS was the only body of the scientific community endowed with any political power [38]. The *Act on the Polish Academy of Sciences* originating in 1952 remained in effect until 1997 and kept the central Academy bureaucracy and seven Secretaries in charge of administrating PAS institutes. An important role was played by the PAS headquarters, as it preserved its privileges and, for many years after the political transformation in 1989, ‘the process of restructuring is hampered by the tactics of those seeking to preserve whatever exists, as if everything that existed deserved preservation, and reasonable preservation were possible without restructuring’ [37].

The *Act on the Polish Academy of Science* passed in April 1997 left the organizational structure of the PAS intact. The new law stated that the PAS was to serve as a corporation for gathering scientists and as an administrative umbrella organization for various research institutes. These two tasks were not clearly separated. Therefore, the powerful position of the president was enforced and no systematic and transparent evaluation of scientific work occurred. Ideas to close down institutes or merge them with other HE institutes or GRDIs were strongly resisted. Both the PAS headquarters and the PAS institutes were ‘interested in increasing their financing and preserving existing structures’ [41]. Thus, it was helpful for the PAS establishment that, in international rankings, PAS institutes typically ranked much higher than either HE or GRDIs. For example, in the SRI World Report (2010) the PAS was ranked at position seventy-two and the first HE institute appears at position 362.

The second explanation is that the PAS was shielded from direct environmental influences via the peer review-based funding mechanism that was introduced in 1991 in tandem with the KBN. As mentioned above, the funding for GRDIs, HE, and the PAS was allocated predominantly in the form of statutory funding. Up to the mid-2000s, the sole distributor of these statutory funds was the KBN. Being partly elected by the Polish scientific community and partly appointed by the government, the KBN gave established Polish scientists considerable influence on funding decisions. Because Poland has a relatively small scientific community, the introduction of peer review-based funding via KBN meant that established networks, loyalties, and perceptions of excellence among scientists influenced funding decisions. We can find a detailed description of the foundation of the KBN, the actors involved in it and the shortcomings it entailed in previous studies by Jablecka [11], [15]. However, relevant to this analysis is the impact of the funding mechanism introduced by the KBN on the organizational structures of the PPSS. In order to determine how this funding mechanism has influenced the performer level, data on funding allocation will be presented and analysed in regard to the three research performing sectors.

Most importantly, the KBN was responsible for the allocation of statutory funding, which covers direct and indirect costs related to the research mission of PAS institutes, HE, and GRDIs. The funding was allocated annually in a competition based on ex-post evaluations made by disciplinary subcommittees, which ranked the institutes from A to D (Table 2). These ranks represent the scientific output in relation to the number of staff and compared to other units in this scientific area. A-ranked research institutes received 100 per cent of their current budget, B-ranked received up to seventy per cent; C-ranked up to fifty per cent; and D-ranked institutes were not eligible for any statutory funding [42].

**Table 2:** Categories assigned in institutional ranking by KBN (1992–2004) and the MNiSW (2007), published in Official Journals [43].

Category	PAS					GRDIs					HE				
	1992*	*2000*	*2004*	2007*	2013*	*1992*	*2000*	*2004*	*2007*	2013*	*1992*	*2000*	*2004*	*2007*	2013*
**“1”/”A”**	61	42	47	68	60	59	27	23	49	72	119	116	105	210	222
**“2”/”B”**	15	26	16	5	2	83	63	56	70	40	178	172	177	155	355
**“3”/”C”**	3	7	7	4	-	61	64	51	29	-	155	155	179	85	22
**“4”/”D”**	3	2	1	-	-	36	39	19	16	-	85	87	44	29	-
**“5”**	-	2	0	-	-	-	23	3	-	-	-	42	13	-	-
**“M”**	-	0	10	-	-	-	0	42	-	-	-	0	42	-	-
**Total**	82	79	81	77	62**	239	216	194	164	112**	537	572	560	479	599**

* in 1992, 2007, and 2013 the A to D categorization was used (in 2013 there was an “A+” which is included in the “A” category here; for further details see text); in 2000 and 2004 the 1 to 5 system, M being a residual category.

**Since research institutes are evaluated every four years, not all institutes are accounted for. The high number of HE institutes is due to the fact that all units at the HE institutions are counted, not only the research institutes.

In the early 1990s, approximately seventy-five per cent of PAS institutes received A rankings, in contrast to twenty-two per cent of HE and twenty-five per cent of GRDIs. However, as seen in Table 2, funding for many PAS institutes plummeted after 1992 because fewer institutes received A grades and more institutes received B and C grades. New criteria and procedures for statutory funding were laid out in regulations passed in November 2001 and incorporated into the *Act on the Principles of Financing Science* passed in October 2004. Following these new rules, fifty-eight per cent of the PAS institutes received a “1”, the equivalent of the former A grade, whereas twenty per cent of the HE institutes and thirteen per cent of GRDIs received such a high funding score. Since the new legal acts passed in 2010/11, every R&D performing unit has undergone a mandatory external audit at least every four years, carried out by the *Committee of Evaluation of Scientific Units* (KEJN), which includes representatives from science, business, and sector ministries. From now on, research units are evaluated in so-called groups of common assessment (GWO) with similar size, type, and scientific profile–as well as two ideal units of references for the “A” and “B” categories. In the first evaluation round in October 2013, in accordance with the new law, twelve PAS units received an A+ rating (leading level), forty-eight received an A (very good level), and only two were marked with a B (level of satisfaction with the recommendation to strengthen the scientific, research and development, or stimulating innovation in the economy); none received a C (unsatisfactory). Units ranked at A+ will receive priority funding; units ranked with a C will receive further funding for six months, providing them with the opportunity to restructure [44]. The fact that the statutory funding lay predominantly in the hands of the scientists themselves raised suspicions of undue advantages and self-interest. The lack of clear accountability for decisions or fear for disregard of non-scientific needs and expectations led to strong criticism, as the president of the PAS himself stated: ‘[&] it is difficult to unreservedly accept a system in which public funds are distributed in accordance with the interests and opinions of one of the stakeholders, even a stakeholder as significant as the scholars themselves, and thereby lacking opportunities to consider the interests of the whole society’ [45].

In summary, the relative stability of the number of PAS institutes, relatively modest reduction in scientific staff compared to GRDIs, and consistently growing R&D budget is explained by a low level of environmental pressures compared to GRDIs and HE. In addition, the KBN funding mechanism shielded the PAS from possible environmental pressures. Certainly, the KBN funding mechanism also worked in the case of GRDIs and HE, where it buffered the impact from changes in the institutional environment: decreased demand for R&D services (GRDI) and increased demand for tertiary education (HE). Thus, it can be concluded that the strong influence of the Polish scientific community on the distribution of statutory funding to the PAS, GRDI, and HE units erected buffers against swift and deep institutional change.

## Conclusion

Since the explanation of abrupt and radical nature of transformations in post-socialist countries is pervasive in the literature, it has rarely accounted for processes of more gradual change. The theories and concepts developed in studies on post-socialism, which are often based on the path-dependency approach and focus on the political and the economic sector, cannot capture all processes of change ongoing in post-socialist societies. Other sectors, like public science, have not undergone the same abrupt change and their analysis calls for a different set of analytical tools. Thus, this paper applied an historical institutionalism approach in order to capture the transformation process more appropriately by studying the nature of change processes which occurred on the performer-level. While previous studies addressed macro-level changes in research policy, including policy learning, this paper identifies the change processes which took place in the organizational structures of the PPSS. These are layering in the case of HE, displacement in the case of GRDIs and reproduction by adaptation as well as displacement in the case of the PAS. These processes are shown in the longitudinal datasets presented here for the first time. The developments in the number of units, staff and expenditures provide supporting and complementary evidence for the findings of previous studies that focused on the policy level [11].

The analysis of the macro-data on the post-socialist PPSS shows that–despite of early legal reforms and the introduction of a new funding scheme–no immediate shutdown or mass layoff occurred. Changes in its formal rules did not immediately affect the organizational structures of the PPSS. An explanation for this discrepancy is offered by the concept of gradual transformation. It stresses that a change in the formal rules need to be a) enacted and b) realigned with changing conditions in the social, political and economic environment. Both the enactment and the realignment differ within the three sectors due to the characteristic organizational capacities of each sector and the specific environment they are embedded in (see Discussion).

As a result, the GRDIs were largely dismantled, in regards to units as well as staff; the HE grew in units, staff, and budget. These differing developments for GRDIs and HE can be explained by the new demands from society and the economy, as well as by their Socialist past. The environmental changes and affects described in the Discussion section are important in understanding why processes of *dismantling* (GRDIs) and *layering* (HE) occurred. Not being affected by these kinds of societal shifts in demand, the PAS was able to *reproduce* its privileged position within the new funding system. Here, the KBN funding mechanism that provided substantial buffers against environmental pressures was particularly effective. The lack of clearly formulated and implemented science policies in the first twenty years, combined with powerful actors striving for stability, has conserved certain socialist legacies, particularly in the case of the PAS. This conservation occurred on various levels, from the administrators in the research institutes to the members of the KBN, which served as the sole regulatory power within the PPSS until the mid-2000s. As Jablecka and Lepori have shown in their study on the public research funding system, the KBN as a centralized organization was similar to its Socialist predecessor thereby stabilizing the inherited system. They describe the early reforms as “infusing new political ideas in an organizational structure largely inherited from the past system” [11]. The empirical data presented in this paper supports this perspective on the post-socialist PPSS, which contrasts the image of radical and abrupt change after 1989.

The introduction of far-reaching reforms passed in 2010/2011 can be understood as the reaction towards the cumulative process of change described in this article. With regard to the funding system and the function of the KBN, the new reforms were designed to fundamentally change the PPSS by terminating the bodies’ role as a de facto sponsor and overcoming centralized decision-making. In 2006, the Ministry of Science and Higher Education (MNiSW) was founded, replacing the KBN and assuming its function of distributing funds, setting research policy, and evaluating scientific performances. In order to decentralize the allocation of funds, two separate funding agencies were created: the *National Centre for Science* (NCN), which is responsible for basic research, and the *National Centre for Research and Development* (NCBiR), which is responsible for applied research. The financing of statutory activities, investments in infrastructure and special programs are still allocated by the Ministry.

In addition to the changes in the funding scheme, other structural deficits have been tackled by the new laws, including the missing link between science and the private sector; the relatively weak international position of the PPSS, especially in the research frameworks of the EU; the variation in quality among research institutes of all sectors; and the apparently unsatisfactory maintenance and utilization of newly built infrastructure [46], [47]. With a *national research program* (KPB) being formulated for the first time since 1989 and a program to select *National Leading Scientific Centres* (KNOW), the Polish Government has taken action with respect to the institutional development of the PPSS more actively than it did before. The government also took initiative in the case of the GRDIs, which have been restructured, incorporated, and partly commercialized in order to keep the best performing institutes government owned. Finally, a new law on higher education is currently in the making, accommodating demographic developments and the shrinkage of the private HE sector, leading to the ‘remonopolization of the system by the tax-based public sector and the gradual decline of the private sector’ [12]. Whether the PAS, which was nicknamed a ‘dinosaur threatened with extinction’ [48], will continue to exist in its inherited form is uncertain in the light of a continuing loss of prestige.

Overall, there are already signals that the reform package in 2010/2011 has already left its mark. While seventy-five per cent of all public funds were distributed as statutory funding and only twenty-five per cent as project funding in 2005, today forty-seven per cent of public funds are distributed as statutory funding (NCN and MNiSW) and forty-four per cent are allocated through project funding (NCBiR) [49]. These indicators hint at a more decentralized project-based funding system that enables research institutes to adjust their managerial and administrative practices.

## Supporting Information

S1 Table Number of R&D units according to PSSI sector. (PDF)

S2 Table Number of R&D staff, according to PSSI sector: in Full Time Equivalents. (PDF)

S3 Table Gross Domestic Expenditure on R&D (GERD), according to PSSI sector: in million PLN, inflation adjusted. (PDF)
