# Supplementary material for: The Gradual Transformation of the Polish Public Science System
Source: PLoS One. 2016 Apr 14;11(4):e0153260. doi: 10.1371/journal.pone.0153260 (PMC4831804; doi:10.1371/journal.pone.0153260)
Supplement: S1 Table — (PDF) [file pone.0153260.s001.pdf]

Appendix Table 1: Number of R&D units according to PSSI sector

Year	PAS	HEI	GRDI
1970	66	0	126
1971	66	0	130
1972	64	0	138
1973	65	0	137
1974	66	0	265
1975	72	0	285
1976	75	0	293
1977	75	0	299
1978	75	0	303
1979	68	0	321
1980	71	0	322
1981	73	0	311
1982	73	0	280
1983	73	0	278
1984	73	0	275
1985	73	0	277
1986	74	0	275
1987	75	0	278
1988	78	0	297
1989	81	0	297
1991	77	80	260
1992	75	85	296
1993	81	76	252
1994	83	88	310
1995	81	90	273
1995	80	104	253
1996	80	104	255
1997	81	104	256
1998	82	114	246
1999	81	115	240
2000	81	114	240
2001	81	121	232
2002	81	119	211
2003	80	128	201
2004	78	128	197
2005	76	143	194
2006	78	147	190
2007	75	150	180
2008	73	195	150
2009	76	194	129
2010	76	212	124
2011	70	197	116
2012	70	210	119
