# Supplementary material for: The Gradual Transformation of the Polish Public Science System
Source: PLoS One. 2016 Apr 14;11(4):e0153260. doi: 10.1371/journal.pone.0153260 (PMC4831804; doi:10.1371/journal.pone.0153260)
Supplement: S2 Table — (PDF) [file pone.0153260.s002.pdf]

Appendix Table 2: R&D staff, according to PSSI sector, in FTE

Year	PAS	HEI	GRDI
1994	7777	29073	28963
1995	8089	34883	30900
1996	7705	39046	27836
1997	7262	40977	26158
1998	7600	45265	25160
1999	7486	42948	23918
2000	7233	41499	23044
2001	6934	42386	21708
2002	6579	43752	20137
2003	6443	44455	19196
2004	6543	45572	18596
2005	5681	44763	17546
2006	5817	41535	16330
2007	5918	42595	15698
2008	5861	43479	14801
2009	5454	41299	13904
2010	6008	43022	14157
2011	6047	44071	14235
2012	6324	42845	14914
