# Supplementary material for: The Gradual Transformation of the Polish Public Science System
Source: PLoS One. 2016 Apr 14;11(4):e0153260. doi: 10.1371/journal.pone.0153260 (PMC4831804; doi:10.1371/journal.pone.0153260)
Supplement: S3 Table — (PDF) [file pone.0153260.s003.pdf]

Appendix Table 3: Gross Domestic Expenditure on R&D, in mln PLN, inflation adjusted

Year	PAS	HEI	GRDI
1994	646.50	1089.36	2558.66
1995	632.38	1335.71	2405.95
1996	631.94	1524.80	2338.89
1997	660.79	1661.31	2402.07
1998	664.40	1713.16	2440.40
1999	718.21	1841.47	2623.27
2000	723.82	1990.00	2460.00
2001	777.06	1982.42	2247.63
2002	718.73	1877.36	2023.87
2003	777.25	1759.12	2000.36
2004	817.88	1938.00	2180.00
2005	860.30	2025.66	2108.29
2006	898.18	2076.02	2229.55
2007	927.27	2508.43	2289.80
2008	985.73	2761.02	2332.69
2009	1027.08	3436.51	2291.18
2010	1233.80	3863.70	2469.50
2011	1202.40	3923.70	2547.60
2012	1226.20	4567.04	2423.52
